# Neglect of Children with Disabilities: A Scoping Review

**DOI:** 10.3390/children12030386

**Published:** 2025-03-20

**Authors:** Siwar Makhoul Khoury, Ayala Cohen, Matteo Angelo Fabris, Ayelet Gur

**Affiliations:** 1 Social Work Department, Research Center for Innovation in Social Work (RCISW), Tel-Hai College, Upper Galilee, North District, Qiryat Shemona 1220800, Israel; ayalac@telhai.ac.il (A.C.); guraye@telhai.ac.il (A.G.); 2 Department of Psychology, University of Turin, 10124 Turin, Italy; matteoangelo.fabris@unito.it

**Keywords:** children, neglect, intellectual disabilities (ID), autism spectrum disorder (ASD), scoping review

## Abstract

**Background:** Children with disabilities face an increased risk of neglect and maltreatment due to their dependence on caregivers, social isolation, and challenges in seeking help. While extensive research has examined child abuse, neglect remains an underexplored yet pervasive issue affecting this vulnerable population. **Objective:** This scoping review synthesizes literature from the past decade to assess the prevalence, characteristics, and risk factors of neglect among children with disabilities, aiming to identify gaps in research and inform policy and intervention efforts. **Methods:** Following the PRISMA-ScR guidelines, a systematic search was conducted across multiple electronic databases, including PsycNET, Social Services Abstracts, ERIC, PubMed, and EBSCO. Studies were included if they focused on neglect among children with disabilities and were published in English within the last ten years. Thematic analysis was employed to extract and categorize findings. **Results:** Sixteen studies met the inclusion criteria, revealing a significantly higher prevalence of neglect among children with disabilities compared to their typically developing peers. The type and severity of disability influenced the likelihood and nature of neglect, with children with intellectual disabilities (ID), autism spectrum disorder (ASD), and sensory impairments facing particularly high risks. Key risk factors included parental stress, economic hardship, limited access to resources, and systemic failures in early identification and intervention. Despite the severity of neglect, evidence-based preventive strategies remain scarce, and existing child protection frameworks often fail to account for the unique needs of children with disabilities. **Conclusions:** The findings underscore the urgent need for targeted interventions, specialized training for professionals, and policy reforms to address the neglect of children with disabilities. Future research should focus on developing and evaluating culturally sensitive and disability-specific support systems to mitigate the long-term consequences of neglect.

## 1. Introduction

Children with disabilities comprise a heterogeneous and vulnerable population, affected by a wide range of neurodevelopmental and physical conditions that can profoundly influence their daily functioning and quality of life. The World Health Organization’s International Classification of Functioning, Disability, and Health (ICF) offers a multidimensional framework for conceptualizing disability. This model moves beyond a purely medical perspective, emphasizing that disability arises from the complex interplay between an individual’s health status, bodily functions and structures, capacity for activities, and societal participation.

The ICF conceptualizes disability as a dynamic construct involving impairments in body functions or structures, limitations in activity performance, and restrictions in social participation. This comprehensive approach underscores the critical role of environmental factors and societal attitudes in shaping the disability experience [1]. Recent global estimates indicate that approximately 16% of the world’s population lives with a disability, with prevalence rates on an upward trajectory due to demographic shifts, enhanced diagnostic capabilities, and medical advancements [1]. The growing prevalence of disability, particularly among children, highlights the pressing need to address the multifaceted challenges faced by this vulnerable group.

Research indicates that children with disabilities are more likely to experience various forms of maltreatment compared to their peers without disabilities. Factors contributing to this heightened risk include their dependency on caregivers, social isolation, and challenges in expressing their needs or seeking help [2,3,4]. Understanding the unique experiences of children with disabilities is crucial for developing effective interventions and support systems that ensure their protection, promote their rights, and foster an inclusive environment where they can thrive.

### 1.1. Child Neglect

Child neglect is the most frequently reported form of maltreatment reported to child protection authorities, and at the same time, it appears to be the least studied form of child maltreatment in the literature [5]. Child neglect is a critical aspect of child maltreatment, but a clear distinction must be drawn between abuse and neglect, as they represent different forms of maltreatment. Abuse involves deliberate acts of harm, such as physical, sexual, or emotional abuse. In contrast, neglect refers to the failure to meet a child’s basic needs, such as food, shelter, education, medical care, or emotional support [1,6]. While both forms of maltreatment are concerning, this review focuses exclusively on neglect as it pertains to children with disabilities. Research indicates that children with disabilities are at an elevated risk of neglect due to their dependence on caregivers, social isolation, and challenges in expressing their needs [7,8]. Research has shown that emotional abuse and neglect are often more prevalent among these populations than other forms of maltreatment [9]. However, findings regarding the prevalence of neglect among children with disabilities remain inconsistent. Some studies suggest that having a disability is a significant risk factor for neglect [10], while others indicate that the relationship may be influenced by socioeconomic variables and the definitions of neglect utilized in research [11]. For instance, studies have found that younger children may experience higher rates of neglect than older children, suggesting that age may play a role in vulnerability [12].

The neglect of children with disabilities is not just a public health concern; it is a fundamental human rights issue. The United Nations Convention on the Rights of Persons with Disabilities (CRPD) explicitly recognizes the rights of individuals with disabilities to live free from neglect, discrimination, and abuse. Article 7 of the CRPD emphasizes the need to protect children with disabilities and ensure their full development and participation in society. Countries that are signatories to the CRPD are obligated to implement measures that prevent neglect, support families, and promote inclusive systems that uphold the dignity and rights of children with disabilities (Committee on the Rights of Persons with Disabilities) [13].

The consequences of child neglect are severe and long-lasting, affecting children’s physical and mental health, development, and overall well-being [14]. Moreover, neglect is not only a challenge for child protection systems but also a broader societal issue that requires urgent attention and intervention [15].

The complexity of child neglect is further compounded by the lack of a universally accepted definition. The wide range of definitions in professional practice and academia impedes the precise measurement and identification of neglect [16,17]. Despite existing mandatory reporting laws, professionals often struggle to define and report cases of neglect, particularly emotional neglect, as it is less visible than physical neglect [18,19].

Neglect is also a dynamic and context-dependent phenomenon. Infants and young children, who are entirely dependent on caregivers, are particularly vulnerable to neglect, which can lead to developmental delays, health issues, and cognitive and emotional difficulties [12]. Adolescents, while more independent, may experience different forms of neglect, such as emotional distress, academic difficulties, exposure to domestic violence, and mental health struggles. Research highlights that neglect in adolescence is associated with delinquency, antisocial behavior, and increased risk-taking behaviors [20].

Given the profound and long-term effects of neglect, there is an urgent need to enhance prevention, identification, and intervention strategies. Han et al. (2024) [21] emphasize the importance of investing in the development of strategies for detection, measurement, prevention, and intervention. Community involvement and a child-centered approach to defining neglect can help address the issue more effectively [22]. Furthermore, understanding the perspectives of children and adolescents regarding neglect is essential for developing effective protective measures and support systems [23].

### 1.2. Research Objective

In light of these complexities, the present study aims to conduct a scoping review of articles published in the last ten years that investigate the issue of neglect among children with disabilities. By synthesizing existing literature, this review seeks to clarify the prevalence, characteristics, and factors associated with neglect in this vulnerable population, thereby identifying gaps in the current understanding and highlighting the need for targeted interventions and policies.

## 2. Methods

### 2.1. Search Strategy

This systematic review was conducted following the guidelines of the PRISMA-ScR (Preferred Reporting Items for Systematic Reviews and Meta-Analyses extension for Scoping Reviews) checklist [24]. To identify articles examining the field of neglect among children with disabilities, we utilized four electronic databases: PsycNET, Social Services Abstracts, ERIC, PubMed, and EBSCO.

To ensure a comprehensive review, multiple search terms were used for each characteristic (disability, child, neglect), allowing us to capture a wide range of relevant articles. The selection of these search terms was informed by recommendations from previous systematic reviews in this field.

For disability, we used:

“disability” OR “disabilities” OR “special needs” OR “intellectual disability” OR “intellectual and developmental disability” OR “intellectually disabled” OR “cognitive disabilities” OR “autism” OR “ASD” OR “developmental disability” OR “developmental disorder” OR “ID” OR “IDD” OR “DD” OR “developmental delay” OR “learning disability” OR “attention deficit disorder” OR “attention deficit hyperactivity disorder” OR “ADHD” OR “learning disabilities” OR “mobile disability” OR “mobility disability” OR “visual disabilities” OR “visual impairments” OR “hearing disabilities” OR “hard of hearing” OR “deaf” OR “hearing impairment” OR “sensory disability” OR “mental disability” OR “mental disorder” OR “mental illness” OR “psychiatric” OR “cerebral palsy” OR “CP” OR “acquired brain injury”.

For neglect, we used:

“neglect” OR “maltreatment” OR “emotional neglect” OR “medical neglect” OR “physical neglect” OR “educational neglect” OR “social neglect” OR “lack of supervision or guidance neglect”.

For child, we used:

“child” OR “children” OR “adolescent” OR “teen” OR “youth” OR “young person” OR “young people”.

The combination of these terms and databases ensured the identification of a broad and diverse set of studies, enabling a thorough exploration of the prevalence, characteristics, and factors associated with neglect among children with disabilities.

### 2.2. Selection Criteria

#### 2.2.1. Inclusion Criteria

Studies were included if they focused on neglect in children with disability. For example, the study by Wang et al. [25] was included because it focused on childhood maltreatment, including neglect and the relationship between physical disability or long-term health problems and depression. There was a limit on the publication date, from the last 10 years. Both quantitative and qualitative studies were included in the review. Finally, only studies published in peer-reviewed scientific journals and written in English were considered.

#### 2.2.2. Exclusion Criteria

Studies were excluded if they did not focus on neglect among children with disability and/or if they included children with ADHD and/or if they were not published in English and/or articles that produced a systematic review of literature and/or gray literature. For instance, a study by Samuel et al. [26] was not included in the review because although it focused on the effects of childhood maltreatment, it included adolescents with ADHD. A study by Legano et al. (2021) [3] was not included in the review because although it focused on the maltreatment of children with disabilities, it was a review of literature.

### 2.3. Selection of Studies

**Figure 1:**
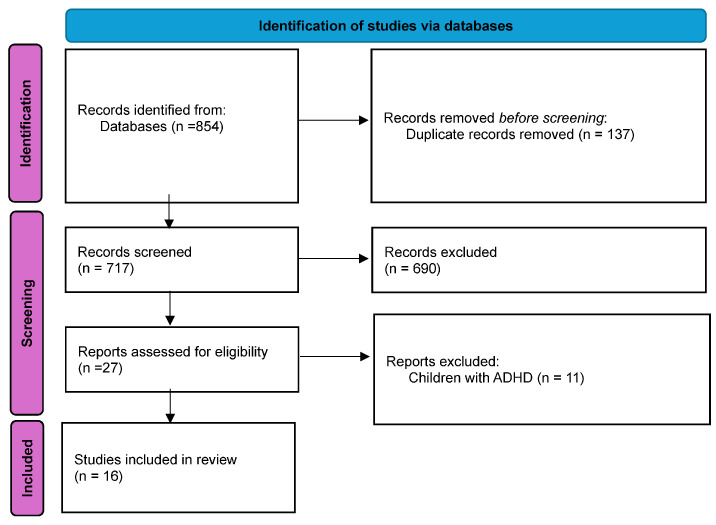
PRISMA flow chart. Review process of articles for inclusion in systematic review (PRISMA flowchart).

The database search yielded 854 articles, which was reduced to 717 after duplicates were removed as shown in Figure 1. The abstracts of the remaining articles were read by two authors who individually determined whether the articles met the criteria of inclusion. Differences in opinion were discussed, and consensus was reached. 27 articles met the inclusion criteria at this point. These articles were fully read by both authors individually, and 11 were excluded because they did not meet the criteria. In the end, 16 articles met the criteria and were included in the review.

### 2.4. Data Coding

Articles were examined for content related to neglect in children with disabilities. To analyze the study results, we employed a thematic analysis approach, which involved a three-step procedure in accordance with Braun and Clarke’s [27] methodology. To begin with, a meticulous line-by-line coding of the papers was conducted. In the subsequent phase, descriptive themes were generated, maintaining a strong alignment with the studies that were incorporated. The third stage involved the creation of overarching analytical themes, going beyond the confines of the study outcomes to establish innovative interpretations [26]. The researchers conducted separate data analysis procedures, during which they individually extracted data from the study results, organized the codes, and pinpointed potential themes. Following this, a collaborative review approach was utilized by the researchers to attain agreement on the identified themes, codes, and principal narratives evident within the data. Ultimately, the researchers classified the core themes to present the findings in a coherent and inclusive manner.

## 3. Results

### 3.1. Characteristics of Articles Included in the Review

Table 1 summarizes the main characteristics of the 16 reviewed articles, including child characteristics, disability, and study design. Studies’ sample sizes ranged from a small sample, such as [28] or 106 participants [26], to the largest sample size of 524,534 participants [29]. One study included only 4 years old children [28], and one study included 180 parents [30]. One study included 106 fatalities [26], and many included deferent disabilities [26,29,31]. Some included children with autism [30,32,33,34,35,36], and two included children that were deaf or hard-of-hearing [37,38].

The studies were conducted worldwide. Four studies were conducted in the United States [26,33,34,36]. Two studies were conducted in Canada [2,4], one in Brazil [38], two in Australia [28,29], three in China [25,30,32], One was conducted in Turkiye [39], one in Japan [35], one in Saudi Arabia [37], and one in Israel [31]. The studies were conducted between 2015 and 2024; six studies were published later than 2020. The earliest included article was from 2015.

### 3.2. Increased Prevalence of Neglect and Abuse Among Children with Disabilities

Table 2 summarizes the main characteristics of the 16 reviewed articles including Aim of study, psychosocial Outcomes, Recommendations and conclusions. The reviewed studies reveal that children with disabilities are at a significantly higher risk of experiencing neglect and abuse compared to their typically developing peers. For example, Karni-Visel et al. (2020) [31] found that the odds of being identified as a suspected victim of maltreatment were 6.2 times higher for children with disabilities than for those without disabilities. Similarly, Fisher et al. (2019) [34] reported that 17.3% of children with ASD had referrals for maltreatment compared to 7.4% of control children, emphasizing the vulnerability of this population.

The prevalence of neglect is particularly concerning. Maclean et al. (2017) [29] demonstrated that children with intellectual disabilities (ID) had a significantly higher likelihood of neglect allegations compared to their peers. Furthermore, children with physical disabilities or long-term health conditions are frequently subjected to neglect and emotional maltreatment, which mediates the development of depression later in life.

### 3.3. Differences Among Disability Types

The type and severity of the disability influence both the likelihood and nature of maltreatment, although findings vary across studies. For example, children with mild or moderate ID are generally more likely to experience maltreatment compared to those with severe ID. However, some studies suggest that the protective factors associated with severe ID might not uniformly apply across different forms of abuse.

In contrast, children with autism spectrum disorder (ASD) alone appear particularly vulnerable to physical abuse, whereas those with comorbid ASD and ID face elevated risks of multiple forms of abuse, including emotional and sexual abuse. While this pattern is widely observed, some research highlights nuances, such as variability in the type of maltreatment based on environmental or familial factors.

Children with sensory impairments, such as those who are deaf or hard-of-hearing (DHH), also exhibit distinct vulnerabilities. Marques et al. (2022) [38] reported that 94.3% of DHH children in their sample experienced both physical and psychological maltreatment, with over 50% enduring severe abuse. Similarly, Hammad et al. (2024) [37] found that almost all DHH adolescents faced moderate to severe maltreatment, which correlated with heightened rates of depression and anxiety. However, other findings indicate differences in the prevalence or severity of maltreatment based on factors such as age, gender, or additional disabilities, suggesting a need for further exploration of this group’s experiences.

### 3.4. Characteristics of Neglect

Children with disabilities face significantly higher risks of neglect, with Karni-Visel et al. (2020) [31] finding they are 6.2 times more likely to be identified as suspected victims of child maltreatment compared to non-disabled peers. The characteristics of neglect vary by disability type and severity; Maclean et al. (2017) [29] reported that children with intellectual disabilities had a higher proportion of neglect allegations (33%), with those having less severe intellectual disabilities showing an almost threefold increased likelihood of maltreatment allegations. Family factors also play a crucial role, as Hammad et al. (2024) [37] found that deaf and hard-of-hearing students from larger families (5+ children) and those with parents having lower education and income levels experienced higher rates of neglect. Samuel et al. (2023) [26] identified that fatalities from neglect were most commonly associated with cerebral palsy, visual impairment, paralysis, and traumatic brain injury, with statistically significant relationships between neglect-caused fatalities and diagnoses of ADHD, ASD, cerebral palsy, and traumatic brain injury. The psychological impact is significant, with Wang et al. (2019) [25] demonstrating that physical and emotional neglect mediated the association between childhood physical disability and depression. Despite these concerning patterns. Keeley et al. (2024) [28] noted a troubling lack of media coverage on the neglect of people with intellectual disabilities, with only 27 articles published over a 5-year period in Australian news media, suggesting limited public awareness of this issue.

### 3.5. Risk Factors

Several factors increase the risk of maltreatment among children with disabilities: Child-related factors: Younger children and boys with disabilities are particularly vulnerable to abuse [31]. Additionally, children with more severe functional impairments or comorbidities face heightened risks [37]. Parental stress and mental health: Parenting stress, especially in the context of caregiving for a child with significant needs, is a well-documented risk factor. Chan and Lam (2016) [32] demonstrated that parenting stress was directly linked to both psychological aggression and physical abuse. Socioeconomic pressures: Economic hardships and parental unemployment are associated with increased maltreatment risk. For example, Hammad et al. (2024) [37] found that children from low-income families with lower parental education levels reported higher rates of maltreatment. Cultural and systemic inequities: In some communities, limited access to healthcare and social services exacerbates the vulnerability of children with disabilities. Karni-Visel et al. (2020) [31] noted that children from minority groups, such as the Arab sector in Israel, were less likely to be identified as victims of abuse due to systemic underreporting.

### 3.6. Protective Factors

Despite the elevated risks, several protective factors mitigate the likelihood of maltreatment for children with disabilities: Parental education and support: Higher levels of parental education and access to resources reduce the incidence of maltreatment. For example, families with access to social support systems, such as healthcare providers and community services, reported fewer instances of neglect [31,37]. Cultural and familial resilience: In some cultures, strong family ties and community networks act as buffers against abuse and neglect. Wang et al. (2019) [25] noted that culturally grounded support systems play a critical role in protecting children with physical disabilities from maltreatment. Formal intervention systems: The involvement of healthcare providers, educators, and child protection agencies in identifying and supporting at-risk families is crucial. Karni-Visel et al. (2020) [31] found that nearly half of the maltreatment reports for children with disabilities were initiated by primary healthcare providers, highlighting the importance of professional vigilance.

## 4. Discussion

The findings of this scoping review highlights the unique vulnerabilities of children with disabilities to neglect and abuse, emphasizing both the complexity and severity of their experiences and the systemic gaps in addressing their needs.

Consistent with previous studies, the findings underscore the heightened risk of neglect among children with disabilities, with prevalence rates significantly exceeding those of their typically developing peers [29,40]. This increased vulnerability can be attributed to the additional caregiving demands posed by disabilities, the socioeconomic challenges faced by many families [4,32,37], and systemic failures in identifying and addressing neglect [26,36].

Children with ASD, ID, and physical disabilities are particularly at risk, with studies reporting that children with ASD are over twice as likely to be referred to child protection services [34]. The findings reinforce the need for targeted interventions to address the specific needs of these subgroups. Moreover, the association between neglect and long-term developmental and psychological harm highlights the urgency of early detection and prevention efforts [32,39].

The review demonstrates that not all disabilities carry the same risk for neglect and maltreatment. For example, children with ID are more likely to experience neglect, while those with ASD often endure multiple forms of abuse, including physical, emotional, and sexual abuse [31]. Similarly, children with sensory impairments, such as those who are deaf or hard-of-hearing, face higher rates of emotional and physical neglect [38].

These differences highlight the importance of tailoring interventions to the unique needs of different disability groups. Policies and services must account for the specific vulnerabilities and support requirements associated with each type of disability [4,37].

A particularly concerning finding is the prevalence of within-family maltreatment, with biological parents often identified as the primary perpetrators. This aligns with research indicating that parental stress, economic hardship, and inadequate support systems contribute to neglect within families [41,42]. The co-occurrence of multiple forms of maltreatment, such as neglect alongside physical or emotional abuse, further exacerbates the challenges faced by children with disabilities [35].

The severity of neglect reported in the findings is particularly alarming, as it often leads to more significant developmental setbacks for children with disabilities. The dependency of these children on caregivers amplifies the impact of neglect, making early intervention critical to mitigating long-term harm [2,26].

The findings illustrate a clear interaction between risk and protective factors in shaping the neglect and maltreatment experiences of children with disabilities. On the one hand, factors such as caregiver stress, financial strain, and systemic failures significantly increase the risk of neglect. On the other hand, access to formal support systems, including respite care and specialized services, can mitigate these risks and improve outcomes for children and their families [43].

The review also highlights the role of community and societal factors in shaping neglect risks. Stigma and discrimination against disabilities often discourage families from seeking help, while systemic inadequacies in child protection services result in underreporting and delayed interventions [44]. Addressing these structural barriers is essential to creating a more inclusive and supportive environment for children with disabilities.

Neglect constitutes a persistent and chronic issue that significantly impacts children’s well-being over time. Its insidious and often imperceptible nature renders it particularly challenging to identify and effectively address [45]. Despite the profound consequences of neglect and its heightened prevalence among children with disabilities, the present literature review found no evidence of targeted interventions designed to prevent or mitigate its effects within this population. Moreover, there is limited evidence on the efficacy of existing interventions, highlighting a critical gap in understanding what approaches are effective in addressing this pervasive issue.

Globally, responses to child neglect remain inconsistent and insufficiently researched. While some countries implement general child protection policies and measures, these frameworks frequently fail to account for the specific vulnerabilities of children with disabilities. Many existing models lack the necessary flexibility to adequately address the needs of neglected children with disabilities, particularly in contexts where chronic neglect has far-reaching and long-term consequences. Furthermore, comprehensive caregiver support systems and early identification mechanisms are scarce, leaving practitioners with limited tools for effective intervention. This absence of structured and evidence-based solutions perpetuates cycles of harm and underscores the urgent need for rigorous research and informed policy development in this domain.

### 4.1. Implications for Policy and Practice

The results of this scoping review have several potential implications for policy and practice: (1) Tailored Interventions: Policies must account for the diverse needs of children with different types of disabilities, ensuring that interventions are disability-specific and culturally sensitive; (2) Strengthening Support Systems: Expanding access to respite care, financial assistance, and community-based programs can reduce caregiver burden and improve child developmental outcomes; (3) Training for Professionals: Child protection workers, teachers, educators, and healthcare providers need specialized training to recognize and address neglect in children with disabilities. Teachers and educators in particular play an important role in recognizing and reporting potential cases of child abuse and maltreatment, as they are the extra-familial caregivers with whom children with disabilities spend the most time [46]. In many countries, professionals dealing with children are obliged to report cases of suspected abuse and maltreatment towards children. This could be done more easily if professionals know how to recognize situations of potential abuse, including neglect [46] Public Awareness Campaigns: Efforts to combat stigma and promote the inclusion of children with disabilities can foster supportive communities and reduce neglect risks.

### 4.2. Research Gaps and Future Studies

While this review provides critical insights, significant gaps remain that warrant further investigation. A key foundational step in addressing these gaps is the establishment of a shared definition of neglect. Neglect can take many forms—physical, emotional, educational, and medical—each with unique characteristics that may be particularly difficult to detect in children with disabilities. Unlike more overt forms of abuse, neglect often presents subtly and insidiously, complicating the ability of professionals to identify and intervene effectively. Therefore, it is essential to explore these different forms of neglect more closely, ensuring a comprehensive understanding that informs both research and practice.

A notable void lies in the lack of evidence-based programs specifically designed to prevent neglect among children with disabilities. This phenomenon, unlike more overt forms of abuse, is often subtle and insidious, leaving professionals feeling powerless to identify and address it effectively. Additionally, most studies rely on cross-sectional designs and small samples, limiting the generalizability of findings. Future research should adopt longitudinal and intersectional approaches to explore the complex dynamics of neglect among children with disabilities. Investigating underrepresented populations, such as children with sensory impairments or rare disabilities, is also essential to ensure that all children receive equitable support.

## 5. Conclusions

Neglect among children with disabilities is a multifaceted issue requiring a multi-pronged approach. The findings of this review underscore the urgent need for targeted interventions, systemic reforms, and increased public awareness to address the unique vulnerabilities of this population. A dedicated effort is required to develop evidence-based programs, train professionals, and establish collaborative frameworks to address neglect effectively. Without these steps, children with disabilities will continue to face disproportionate risks, while professionals remain unequipped to provide meaningful support. By bridging research gaps and implementing inclusive policies, we can work toward ensuring that all children, regardless of ability, receive the care and support they deserve.

## Figures and Tables

**Table 1:** Characteristics of articles included in the review: child characteristics, disability, and study design.

**Reference No.**	**Year**	**Country**	**Participants**	**Child Characteristics**	**Disability**	**Study Design**
[32]	2016	Hong Kong China	424 children & parents	86.1% parents Average age 43.60 years 16.7% children Children 10.4 years	ASD	Quantitative research
[2]	2018	Canada	5797 children	0–14 years	Intellectual disability	Analysis of cross-sectional dataset
[30]	2015	China	180 parents	2–5 years children 138 boys, 42 girls. Parents: 41 fathers aged 34.5, 139 mothers aged 32.5	Autism	Cross-sectional study
[34]	2019	Tennessee, USA	387 children	10 years of age. Have ASD	Autism spectrum disorder	Report
[37]	2024	Saudi Arabia	186 children	Aged 14–17 years (M = 15.7 years; SD = 3.41 years)	Deaf and hard-of-hearing	Quantitative research
[35]	2021	Japan	378 children	Age 6 to 18. Under institutional care. 45.2% girls, mean [SD] age = 12.6 [3.4] years	ADHD Autism spectrum	Quantitative research
[31]	2020	Israel	56,816 children	Children without disabilities (53,739) or those with a developmental disability (3077) 49.5% boys, with a mean age of 6.7 years The age range was composed of 41% up to 5 years old, 37% between 6 and 10 years old, and 22% from 11 to 15 years old. More than half were Arabs (57%), 34% were from the general sector, and 9% were Haredi (Ultra-Orthodox Jews)	Having a medical record with a developmental diagnosis (e.g., autism, intellectual disability, ADHD; developmental language disorder; developmental coordination Disorder), received a series of developmental treatments (occupational therapy, physiotherapy, language therapy)	Cohort study
[28]	2024	Australia	27 reports	4-years-old	Intellectual disability	Foucauldian discourse analysis
[29]	2017	Western Australia	Children born between 1990 and 2010	524,534 children in the population cohort. 4.6% had a maltreatment allegation. Overall, 25.9% of child maltreatment allegations and 29.0% of substantiated allegations involved a child with a disability	Intellectual disability Down syndrome Birth defects/cerebral palsy (all congenital malformations and cerebral palsy) Autism Conduct disorder Mental and behavioral disorder	Population-based record-linkage study
[38]	2022	Maceió, Northeast Brazil	265 children	240 (90.6%), moderate hearing loss. 25 severe hearing loss. 189 (71.3%), were male, and 157 (59.2%) were under 10 years of age	Deaf or hard-of-hearing	Cross-sectional study
[33]	2019	USA	789 children	2–17 years 700 children not maltreated 80 children neglected. 89 experienced neglect and/or physical or sexual abuse	Autism	Analyses conducted retrospectively on existing clinical record
[36]	2019	South Carolina	4988 children	1992, 1994, 1996, and 1998. Children with ASD-only (*n* = 316), ASD+ID (*n* = 291), and ID-only (*n* = 1280)	ASD ASD+ID ID	Using record linkage between the Department of Social Services (DSS) and the Autism and Developmental Disabilities Monitoring (ADDM) network
[4]	2018	Canada	1012 children	62 with ID	Intellectual disabilities	Secondary use of data derived
[26]	2023	USA	106 fatalities	Average age 5.9 years 74.6% were male	Attention deficit hyperactivity disorder, autism spectrum disorder, cerebral palsy, and/or traumatic brain injury	Retrospective analyses
[39]	2024	Türkiye	196 children 98 with SLD 98 control group	60 males, 38 females 9.16 average age of child with SLD, 9.52 average age of control group	Specific learning disorders (SLDs)	Single-center cross-sectional case–control study
[25]	2019	China	5726 children	Middle and high school students aged 12–18 years old	Physical disability	Quantitative research

**Table 2:** Characteristics of articles included in the review, aim of the study, psychosocial outcomes, recommendations, and conclusions.

**Reference No.**	**Year**	**Aim of Study**	**Psychosocial Outcomes**	**Recommendations and Conclusions**
[32]	2016	To examine different individual and environmental influences as potential risk factors for harsh discipline among parents of children with ASD. To examine the link between psychological aggression and physical assault.	Child symptom severity, parenting stress, family economic pressure, and experienced discrimination were positively associated with parental psychological aggression. Child symptom severity and parenting stress were positively associated with parental physical assault. Parenting stress continued to be significantly linked to psychological aggression, while child symptom severity and parenting stress explained unique variance in physical assault. The effect of parenting stress on physical assault was partially mediated by psychological aggression.	Future child maltreatment intervention programs should consider teaching parents appropriate ways to respond to their children with ASD and manage their children’s atypical behaviors.
[2]	2018	To gain a better understanding of maltreatment situations of children with ID. To compare maltreated children with ID to those without ID in terms of their characteristics and characteristics of their caregivers, the substantiated child maltreatment report, and child protection service provision.	Functional problems were higher among children with ID and their parents. Children with ID experienced more severe maltreatment and were more often referred to ongoing child protection services	Maltreated children with ID are facing additional challenges that must be accounted for in service planning and delivery.
[30]	2015	To investigate the prevalence of child physical maltreatment in children with autism. To explore the risk factors for severe child physical maltreatment in the children with autism	Child physical maltreatment was widespread in families of children with autism.	The reality that autism is associated with child physical maltreatment probably needs to be addressed in all these areas with better services, more provision of respite, and support and training to help the parents cope with any issues without resorting to child physical maltreatment.
[34]	2019	To rate maltreatment referrals, screening for further action. To examine substantiated maltreatment for children with versus without autism spectrum disorder.	Children with autism spectrum disorder were less likely than children without autism spectrum disorder to have referrals screened. Maltreatment rates were similar across groups. Girls versus boys with autism spectrum disorder were more likely to have substantiated maltreatment.	Ultimately, it is vitally important for states to know the proportion of children within their child protection systems who have ASD in order to develop systems of care inclusive of effective maltreatment prevention, response, assessment, and intervention strategies.
[37]	2024	To determine the prevalence of child maltreatment and to examine its association with depression and anxiety among a sample of deaf and hard-of-hearing students in Saudi Arabia,	About 47.3% of the students were exposed to severe to very severe child maltreatment. The severity of maltreatment varied based on parents’ educational and income level, number of children in the family, the deaf and hard-of-hearing student’s gender, and parents’ hearing status. Child maltreatment was a significant predictor of depression and anxiety in this sample.	It is important to work with parents of deaf and hard-of-hearing children to improve their skills in rearing a child with special needs. Addressing the social stigma and social barriers experienced by DHH individuals through familial, institutional, and community interventions. It is important to recognize parental abuse among DHH children and adolescents as a public health concern and to develop appropriate strategies to prevent the same.
[35]	2021	To investigate the severity of ADHD and ASD symptoms in institutionalized children. To examine the associations of each type of maltreatment with the severity of ADHD and ASD symptoms. To analyze the effect of hyperactivity/impulsivity symptoms and inattentive symptoms separately to determine how each symptom was related to being maltreated.	ADHD and ASD symptoms were frequent in institutionalized children, and the symptoms of ADHD and ASD were associated with the experience of being maltreated. Maltreatment experience before being institutionalized was associated with hyperactive/impulsive symptoms and inattentive symptoms but not with autistic traits. Physical abuse was associated with hyperactive/impulsive symptoms and autistic traits but not with inattentive symptoms. Psychological abuse was associated with hyperactive/impulsive symptoms. Neglect was not associated with inattentive symptoms, hyperactive/impulsive symptoms, or autistic traits.	There is a need for awareness of the range of issues faced by these children and the necessity for interagency collaboration to ensure that their complex needs are addressed. Support is required for families with children with disabilities to assist in meeting the child’s health and developmental needs, as well as to support parents in managing the more complex parenting environment.
[31]	2020	To explore primary care providers reporting rates of suspected child maltreatment among children with disabilities vs. children without disabilities attending community health clinics.	Health professionals identified CM in those with a disability at a higher rate than those without a disability.	Support is necessary for families with children with disabilities to help address the child’s health and developmental needs but also to assist parents in managing the more complex parenting environment.
[28]	2024	To develop an understanding of the narratives that are constructed by and influence public perceptions of people with intellectual disabilities within the context of familial neglect.	The subject positioned construct people with intellectual disabilities as different and vulnerable and presented limited consideration of suffering. The lack of news coverage hindered opportunities for people with intellectual disabilities to participate equally in society.	Social attitudes (presented through the media) about people with intellectual disabilities within the context of family neglect demonstrate social inequality and require change.
[29]	2017	To report the prevalence of different disabilities within the child protection system in an Australian state. To assess the risk of maltreatment in various types of disabilities, taking into account child, family, and neighborhood risk factors.	Children with disabilities make up 10.4% of the population; they represent 25.9% of children with a maltreatment allegation and 29% of those with a substantiated allegation. Children with intellectual disabilities, mental/behavioural problems, and conduct disorders continued to have an increased risk of an allegation and substantiated allegation after adjusting for child, family, and neighborhood risk factors.	The prevalence of disabilities in the child protection system suggests a need for awareness of the scope of issues faced by these children and the need for interagency collaboration. Support is needed for families with children with disabilities to assist in meeting the child’s health and developmental needs but also to support the parents in managing the often more complex parenting environment.
[33]	2019	To determine if autism symptoms differed between children with autism who were and were not maltreated and if the duration of and number of years since removal from neglect were related to symptoms.	Symptoms of autism were likely independent of maltreatment.	Autism in maltreated children should not be considered as “quasi-autism” or possibly temporary because these children may then be denied. Evidence-based intervention that can improve outcomes in children with autism.
[36]	2019	To evaluate the odds of experiencing maltreatment among children with ASD and/or ID in comparison to population controls.	All groups were more likely to experience reported and substantiated physical neglect. Children with ASD+ID and ID-only were more likely to experience reported sexual, physical, and emotional abuse, whereas children with ASD-only were only more likely to experience reported physical abuse.	There is an urgent need for empirically supported assessment and intervention approaches to identify and address trauma-related stress associated with abuse in ASD.
[38]	2022	To verify the frequency of physical assault and psychological aggression, and associated factors experienced by DHH children living in low socioeconomic settings.	221 children (83.4%) experienced physical assault; 238 (89.8%) experienced psychological aggression. Both physical and psychological aggression were reported for 94.3%. Most mothers (98.1%) reported using nonviolent discipline. Maltreatment was associated with male children, mothers’ job dissatisfaction, religiously nonobservant families, and children born of unintended pregnancy.	There is a need for efforts to address this important problem in low socioeconomic settings. Raising awareness of possible child maltreatment among certain populations of DHH children and developing strategies to reduce maltreatment are needed. It is important to reduce social isolation and the stigma associated with DHH children, to educate caregivers on issues related to deafness, and to provide significant community and educational resources.
[4]	2018	To identify the individual, environmental, and service-related factors that distinguish maltreated children with ID from those without ID.	Neglect was the most common form of substantiated maltreatment of children with ID.	Children with disabilities do not receive the attention to which they are entitled, in terms of public policy, research, and practice.
[26]	2023	To identify and characterize child maltreatment-related deaths among CWD using surveillance data, including case narratives. To define the relationship between suspected perpetrators and victims.	Statistically significant relationship between fatalities caused by neglect and diagnoses of attention deficit hyperactivity disorder, autism spectrum disorder, cerebral palsy, and/or traumatic brain injury. Physical abuse and/or neglect resulting in a fatality among children with disabilities were significantly correlated with the relationship of the perpetrator to the victim.	It is important to address educating CWD who have the capacity to understand how to seek help if they are being abused or neglected.
[39]	2024	To compare the rates of neglect and abuse exposure between children diagnosed with SLDs and those with typical development.	Children with SLDs more often came from families with lower parental education levels and were more likely to experience physical and emotional abuse, particularly from peers and family members. The presence of extended family was more common in the SLD group, and over half of these children also had ADHD. No significant difference between the groups in terms of neglect.	Both clinical and educational interventions from the early childhood period would be beneficial for children and for raising awareness in children’s social circles about SLDs in light of the finding that these children were abused by individuals who were close to them and by their peers.
[25]	2019	To identify whether childhood maltreatment mediated the relationship between physical disabilities or long-term health problems and depression.	There were significant differences in childhood maltreatment and depression between adolescents with childhood physical disabilities or long-term health problems and those without. Physical abuse, sexual abuse, emotional abuse, physical neglect, and emotional neglect mediated the association between childhood physical disabilities or long-term health problems and depression.	More attention is needed to be paid to adolescents who experience emotional abuse. To improve the mental health and well-being of children with physical disabilities, we should attach importance to identifying those children who experience childhood maltreatment and provide them with appropriate help.
